# Panretinal photocoagulation plus intravitreal conbercept for diabetic retinopathy in real world: a retrospective study

**DOI:** 10.1186/s12886-023-03157-z

**Published:** 2023-10-04

**Authors:** Xin Wang, Jing Yao, Shengen Li, Wenyi Zhang, Lijun Wang, Aiyi Zhou

**Affiliations:** Department of Ophthalmology, The Second Affiliated Hospital of Xi’an Jiaotong University, Xi’an, 710004 China

**Keywords:** Diabetic retinopathy, Panretinal photocoagulation, Conbercept, Risk factors

## Abstract

**Background:**

To evaluate outcomes of panretinal photocoagulation (PRP) plus intravitreal conbercept (IVC) for diabetic retinopathy (DR) in real world and explore risk factors for patients with poor reactivity and presence of vision-threatening complications after combination treatment.

**Methods:**

Retrospective review of DR patients received PRP plus IVC over 6 months. The main outcome was improvement ≥ 2 steps in ETDRS diabetic retinopathy severity scale (DRSS) levels. Different strategies for eyes receiving IVC within or over 1 month after PRP were analyzed. For patients with DRSS improvement < 2 steps and presence of vision-threatening adverse events, a binary logistic regression method was used to select risk factors.

**Results:**

Sixty one eyes were involved in this study. After treated with combination therapy with a median number of 3 injections, 44% of eyes improved ≥ 2 steps in DRSS levels. A total of 14 eyes (23%) occurred vision-threatening adverse events. No significant difference was found in eyes receiving conbercept within or over 1 month after PRP. Duration of diabetes (OR 0.849, 95%CI 0.734–0.982, *P* = 0.027), GFR (OR 0.961, 95%CI 0.933–0.990, *P* = 0.010) and baseline DRSS levels (OR 3.290, 95%CI 1.483–7.295, *P* = 0.003) were independent risk factors for DRSS improvement < 2 steps after treatment. Occurrence of vision-threatening complications was only related to high DRSS levels (OR 3.668, 95%CI 1.710–7.868, *P* = 0.001).

**Conclusions:**

The combination therapy was effective for most patients with DR in real world. Eyes received PRP combined with earlier or later conbercept was demonstrated no significant difference for outcomes. For patients with poor renal function, high DRSS levels or occurred DR at the early stage of diabetes, follow-up should be strengthened.

## Introduction

Diabetic retinopathy (DR) is the most common microvascular complication of diabetes and is considered the major reason of blindness in working-aged people [1]. The global number of people with DR and vision-threatening DR (VTDR) was estimated to be 103.12 million, 28.54 million, respectively, in 2020. It is projected that there will be 160.50 million people with DR and 44.82 million people with VTDR in 2045 [2].

Panretinal photocoagulation (PRP) has been the gold standard therapy for proliferative diabetic retinopathy (PDR) for more than 4 decades since the Diabetic Retinopathy Study was published [3]. It can improve the state of retinal ischemia and effectively inhibit neovascularization (NV) by destroying the peripheral retina but retaining the central vision [4]. All patients with severe nonproliferative diabetic retinopathy (NPDR) or worse are considerately to receive PRP treatment currently [5]. However, adverse effects of PRP such as increasing risk of macular edema, losing peripheral or night vision function, are widely recognized [6].

Although anti–vascular endothelial growth factor (VEGF) therapy has been proved to be noninferior to PRP in the treatment of PDR [7, 8], repeated intravitreal injections of anti-VEGF agents do carry risks such as acute bacterial endophthalmitis and decrease the cost-effectiveness of the treatment [9, 10]. Recently, the efficacy of anti-VEGF agents as an adjunct therapy to PRP has been showed for patients with PDR. Compared to conventional PRP, patients had a higher improvement in best-corrected visual acuity (BCVA) and thinner central macular thickness (CMT) in combination group. Besides, the combination therapy is thought to increase the rate of success of PRP in regression of NV and need less rescue treatment for DME and vitrectomy [11–13].

Currently, the main anti-VEGF drugs that were used widely in clinical practice included ranibizumab, bevacizumab, aflibercept and conbercept. Ranibizumab and bevacizumab were recombinant humanized monoclonal anti-body fragment which can blind to VEGF-A isoforms, and aflibercept was a fusion protein that trapped VEGF-A, VEGF-B and PIGF. All of them were proved to be effective for DR patients in some prospective studies [14]. Conbercept was a new anti-VEGF drug produced in China which was a recombinant fusion protein with the same target as aflibercept. However, limited studies reported the effectiveness of conbercept in the real world. Therefore, the purpose of this study was to summarize the outcomes of PRP plus conbercept for DR in real clinical practice. The primary outcome was the proportion of eyes improving ≥ 2 steps in the DRSS levels. Secondary outcomes were the change in BCVA and CMT, and NV reduction. The presence of adverse events was assessed for the safety of combination therapy. Besides, for patients with poor response to treatment, risk factors were also explored.

## Methods

This was a retrospective study to assess the effectiveness of PRP plus conbercept for patients with DR (DRSS levels 47–71,75) between June 2018 and December 2021 at the Second Affiliated Hospital of Xi’an Jiaotong University. The study was approved by the ethics committee of the Second Affiliated Hospital of Xi’an Jiaotong University and adhered to the tenets of the Declaration of Helsinki. The informed consent was exempted by the medical ethics committee of the Second Affiliated Hospital of Xi’an Jiaotong University due to the retrospective nature of this study.

Patients received complete standard PRP (according to the Study Treatment Procedure, which was based on the DRS Study [1982]) immediately after diagnosed as DR and intravitreal injection of conbercept (10 mg/mL, 0.5 mg/0.05 ml) was given after PRP. Standard PRP treatment was defined as a total 1200–1600 burns and exposure time of 0.1 s. Laser parameters must have been adjusted to obtain mild white laser burn, with a spot size of approximately 500 µm and separated 1 burn apart between them. The whole process of PRP was completed in 3–4 times in a week. Treatment of conbercept injection was mainly according to the occurrence of NV and DME. A further injection would be given to the patient if NV or DME existed persistently or recurred after a short-term regression. There must be at least one month between two consecutive injections.

### Eligibility criteria

Inclusion criteria: (1) Patients ≥ 18 years old; (2) Type 1 or type 2 diabetes; (3) Moderately severe NPDR to high risk PDR (DRSS levels 47–71,75); (4) Received standard PRP and IVC injection; (5) IVC was given after standard PRP; (6) Had a last visit over 6 months after the first treatment.

Exclusion criteria: (1) Gestational diabetes; (2) History of ocular surgery (including cataract, scleral buckle or any intraocular surgery) within prior 6 months; (3) Other ocular condition that might alter visual acuity or induce retinal NV such as retinal vein or artery occlusion, uveitis or neovascular glaucoma; (4) Ocular media of insufficient quality to obtain the examination images.

### DR grading criteria

**Fig. 1:**
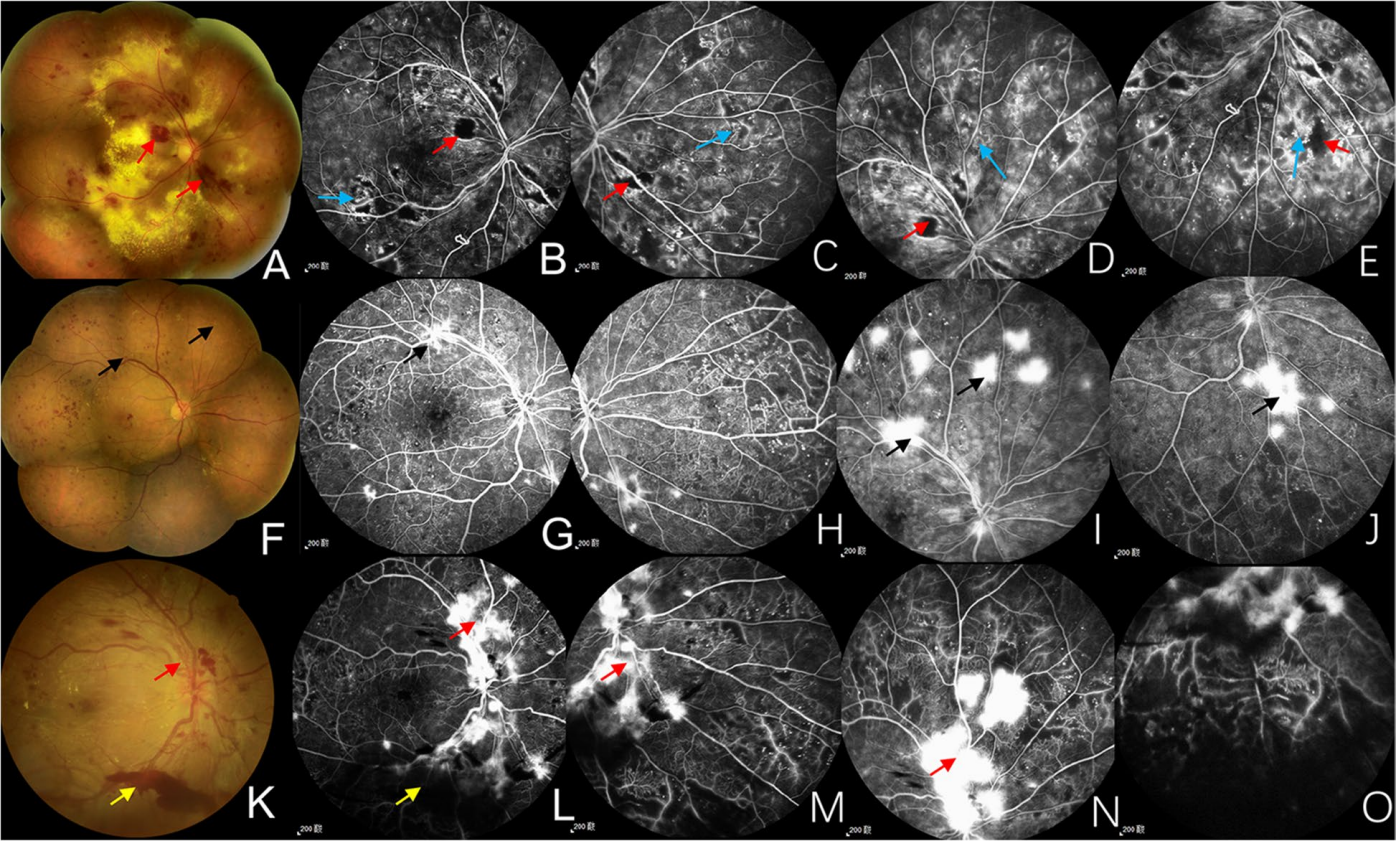
The diagram of DRSS. **A**-**E** DRSS level 53 (very severe NPDR): severe RH (red arrows in FP and FFA) and IRMA (blue arrows in FFA) in 4 quadrants. **F**-**J** DRSS level 65 (moderate PDR): NVE (black arrows in FP and FFA) ≥ 0.5 DA in 1 + quadrants. **K**–**O** DRSS level 71,75 (high-risk PDR): larger NVD and NVE (red arrows in FP and FFA) with PRH (yellow arrows in FP and FFA). FP, fundus photography; FFA, fundus fluorescein angiography; RH, retinal hemorrhage; IRMA, intraretinal microvascular abnormality; NVE, neovascularization elsewhere; DA, disc area; NVD, neovascularization of the disc; PRH, preretinal hemorrhage

The severity of DR was evaluated according to fundus photography (FP), fundus fluorescein angiography (FFA), or a combination of these (Fig. 1). ETDRS diabetic retinopathy severity scale (DRSS) was used for the grading scale of DR, which included 10 levels, from level 10 (no retinopathy) to level 81,85 (advanced PDR). Level 10 (no retinopathy) absented the changes associated with DR. Only the presence of microaneurysms belonged to level 20 (very mild NPDR). Besides microaneurysms, the appearance of hard exudate, cotton wool spot or mild retinal hemorrhage was level 35 (mild NPDR). Level 43 (moderate NPDR), level 47 (moderate severe NPDR) and level 53 (severe NPDR or very severe NPDR) were distinguished from the area and severity of retinal hemorrhage, intraretinal microvascular abnormality or venous beading. The classification of level 61 (mild PDR), level 65 (moderate PDR) and level 71,75 (high-risk PDR) was based on the area of neovascularization (NV). Besides, the level 71,75 may have vitreous hemorrhage (VH) or preretinal hemorrhage (PRH). If the view was partially obscured by VH or PRH from NV, or retinal detachment involving macula occurs, it reached level 81,85 (advanced PDR) [15].

### Data collection

Baseline characteristics included age, gender, body mass index (BMI), duration and family history of diabetes, blood pressure, glycated hemoglobin (HbA1c) and biochemistry. Intraocular pressure (IOP), best-corrected visual acuity (BCVA), central macular thickness (CMT), DR severity scale and presence of DME at baseline were also recorded.

The primary outcome was the proportion of eyes improving ≥ 2 steps in the DRSS levels. Secondary outcomes were the numbers of eyes with NV reduction in patients with PDR, changes in BCVA and CMT, the number of conbercept injections and presence of adverse events. Besides, complications related to treatment were also assessed. Regression of NV was defined as any decrease in the area of NV. DRSS levels improvement ≥ 2 steps from baseline to the last visit was defined as better reactivity to the treatment and < 2 steps was defined as poor reactivity.

### Statistical analysis

Analysis was performed with SPSS software version 23. Normal continuous variables were presented as mean ± SD and median (*P*25, *P*75) was used for non-normally distributed continuous data. Categoric variables were expressed with frequencies and percentages. Visual acuities were converted to logarithm of minimal angle of resolution (logMAR) for analysis. The normality of continuous variable was checked using Shapiro–Wilk test. Student t test was used to compare parametric data and Mann–Whitney U test was used for nonparametric data. Categorical variables were compared using chi-squared test or Fisher's exact test.

A binary logistic regression method was performed to evaluate risks factors. Univariate logistic regression analysis was performed to select the candidate factors with criteria of *P* < 0.05. Factors found significant in the univariate logistic regression analysis were included in the multivariable logistic regression model. *P* < 0.05 was considered statistically significant.

## Results

### Baseline characteristics

A total of 61 eyes (39 patients) with a final visit more than 6 months after PRP plus IVC were analyzed. More than half of patients (64%) were male and the average age of patients was 53.7 ± 12.1 years. Nearly all patients (95%) had diabetes mellitus (DM) of type 2. Systemic baseline characteristics were summarized in Table 1.

**Table 1:** Systemic baseline characteristics

Characteristics	Combination group
No. of eyes	61
No. of patients	39
Gender, n (%)	
Male	25 (64)
Female	14 (36)
Race, n (%)	
Chinese Han nationality	39 (100)
Age, mean (SD), y	53.7 (12.1)
BMI, mean (SD), kg/m^2^	25.4 (4.3)
Diabetes type, n (%)	
Type 1	2 (5)
Type 2	37 (95)
Duration of diabetes, mean (SD), y	12.6 (6.7)
Existence of family history of diabetes, n (%)	9 (23)
Treatment of diabetes, n (%)	
Dietary management	1 (3)
Insulin	15 (38)
Oral hypoglycemic drugs	7 (18)
Combination	16 (41)
Hypertension, n (%)	24 (62)
Hyperlipidemia, n (%)	15 (38)
Diabetic nephropathy, n (%)	21 (54)
Diabetic peripheral neuropathy, n (%)	19 (49)
Diabetic peripheral vasculopathy, n (%)	18 (46)
Medication history, n (%)	
Hypoglycemic drugs	23 (59)
Hypotensive drugs	21 (54)
Antihyperlipidemic drugs	19 (49)
MAP, M (*P*25,* P*75), mmHg	96.3 (86.7, 113.0)
HbA1c, M (*P*25, *P*75), %	9.2 (7.3, 10.2)
TC, mean (SD), mmol/l	4.7 (1.4)
HDL, M (*P*25, *P*75), mmol/l	1.2 (1.0, 1.4)
LDL, mean (SD), mmol/l	3.1 (1.0)
TG, M (*P*25, *P*75), mmol/l	1.6 (1.1, 2.3)
ALT, M (*P*25, *P*75), U/l	16.5 (12.0, 24.3)
AST, M (*P*25, *P*75), U/l	19.0 (16.0, 27.0)
ALP, M (*P*25, *P*75), U/l	80.5 (66.8, 94.8)
GFR, M (*P*25, *P*75), ml/min/1.73m^2^	106.5 (91.1, 122.6)

*BMI* body mass index, *MAP* mean arterial pressure, *HbA1c* glycated hemoglobin, *TC* total cholesterol, *HDL* high density lipoprotein, *LDL* low density lipoprotein, *TG* triglyceride, *ALT* glutamic pyruvic transaminase, *AST* glutamic oxaloacetic transaminase, *ALP* alkaline phosphatase, *GFR* glomerular filtration rate

The median BCVA was 0.5 (0.3, 0.6) logMAR and the mean IOP was 16.6 ± 3.0 mmHg before the treatment. Most of eyes (75%) existed DME before treatment and the median CMT was 276.0 (230.2, 383.8) μm. The proportion of patients with NPDR and PDR were 56%, 44%, respectively (Table 2).

**Table 2:** Ocular baseline characteristics

Characteristics	Combination group
DRSS levels, n (%)	
47 (moderately severe NPDR)	5 (8)
53 (severe NPDR and very severe NPDR)	29 (48)
61 (mild PDR)	10 (16)
65 (moderate PDR)	13 (21)
71,75 (high-risk PDR)	4 (7)
BCVA, M (*P*25, *P*75), logMAR	0.5 (0.3, 0.6)
IOP, mean (SD), mmHg	16.6 (3.0)
CMT, M (*P*25, *P*75), μm	276.0 (230.2, 383.8)
Presence of DME, n (%)	46 (75)

*DRSS* diabetic retinopathy severity scale, *NPDR* nonproliferative diabetic retinopathy, *PDR* proliferative diabetic retinopathy, *BCVA* best-corrected visual acuity, *IOP* intraocular pressure, *CMT* central macular thickness, *DME* diabetic macular edema

### Outcomes of combination therapy in real word

With a median follow-up time of 11.2 (7.1–18.6) months after the combination treatment, the eyes were given to a median number of 3.0 injections. For primary outcome, 27 (44%) eyes improved more than 2 steps in the DRSS levels. For secondary outcomes, the proportion of eyes who gained ≥ 0.1 logMAR in BCVA and decreased ≥ 50 μm CMT were 31%, 23%, respectively. Nearly half of eyes with PDR at baseline had a regression of NV (Table 3).

**Table 3:** Clinical outcomes after combination therapy

Outcomes	Combination group
Primary outcome	
Improvement in DRSS levels, n (%)	
≥ 2 steps	27 (44)
< 2 steps	34 (56)
Secondary outcomes	
NV reduction, n (%)	13 (48)
BCVA gain ≥ 0.1 logMAR, n (%)	19 (31)
CMT decrease ≥ 50 μm, n (%)	14 (23)

*DRSS* diabetic retinopathy severity scale, *NV* neovascularization, *BCVA* best-corrected visual acuity, *CMT* central macular thickness

Regrading safety of combination therapy, there was no serious complications related to treatment in our study (Table 4). A total of 14 eyes had vitreous hemorrhage or preretinal hemorrhage of which 6 eyes developed vitrectomy and 1 eye occurred tractional retinal detachment (TRD).

**Table 4:** Adverse events after combination therapy

Events	Combination group
Vision-threatening adverse events, n (%)	
VH or PRH	14 (23)
Vitrectomy	6 (10)
TRD	1 (2)
Common adverse events, n (%)	
Conjunctival congestion	6 (10)
Conjunctival hemorrhage	2 (3)
Other complications, n (%)	
Iritis	1 (2)

*VH* vitreous hemorrhage, *PRH* preretinal hemorrhage, *TRD* tractional retinal detachment

### Subgroup analysis

For DR patients combined with DME (46 eyes), eyes with NPDR (29 eyes) had a higher improvement in DRSS levels (*P* = 0.003) and a lower occurrence of adverse events (*P* < 0.001) compared to PDR (17 eyes). There was no significant difference in BCVA (*P* = 0.068) and CMT change (*P* = 0.319). Both 3 injections of conbercept were in NPDR and PDR group for patients with DME (*P* = 0.474). However, no significant difference was found between NPDR (5 eyes) and PDR group (10 eyes) for patients without DME (15 eyes) which may due to limited sample size for statistical analysis. The median number of IVC for NPDR and PDR in non-DME group were 1 and 2 injections (*P* = 0.135) (Table 5).

**Table 5:** Outcomes of eyes with NPDR or PDR

Outcomes	non-DME			DME		
	NPDR	PDR	*P* value	NPDR	PDR	*P* value
Improvement in DRSS levels, n (%)						
≥ 2 steps	1	2	1.000	20	4	0.003**
< 2 steps	4	8		9	13	
BCVA change, M (*P*25, *P*75), logMAR	0.0 (0.0–0.0)	0.0 (0.0–0.1)	0.358	0.0 (-0.3–0.0)	0.0 (-0.1–0.5)	0.068
CMT change, M (*P*25, *P*75), μm	76 (-7.5–143.8)	2.0 (-1.5–22.5)	0.642	-19.5 (-117.0–87.8)	29.5 (-63.3–80.5)	0.319
Number of anti-VEGF injections, M (*P*25, *P*75)	1.0 (1.0–1.5)	2.0 (1.0–2.3)	0.135	3.0 (2.0–5.0)	3.0 (2.0–4.0)	0.474
Presence of adverse events, n (%)	2 (40)	4 (40)	1.000	0 (0)	8 (47.1)	< 0.001***

*DME* diabetic macular edema, *NPDR* nonproliferative diabetic retinopathy, *PDR* proliferative diabetic retinopathy, *DRSS* diabetic retinopathy severity scale, *BCVA* best-corrected visual acuity, *CMT* central macular thickness

^*^ *P* < 0.05; **P < 0.01; *** *P* < 0.001

### Early vs late combination treatment

**Fig. 2:**
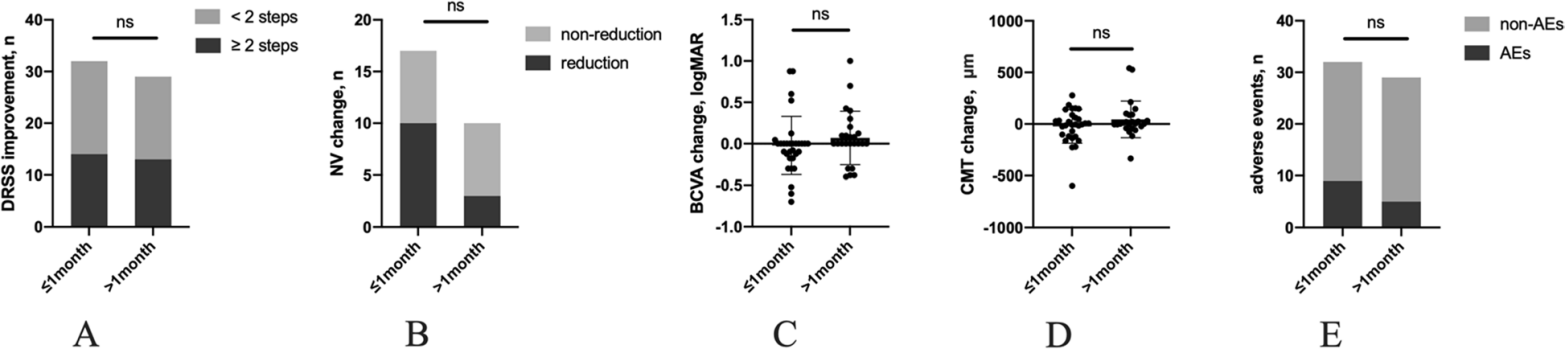
Outcomes of eyes (*n* = 61) treated with IVC within/over 1 month after PRP. **A** The number of eyes with DRSS improvement (*P* = 0.933^a^). **B** The number of eyes diagnosed as PDR (*n* = 27) with reduction of NV (*P* = 0.236^b^). **C** Changes in BCVA from baseline to the last visit (*P* = 0.078^c^). **D** Changes in CMT from baseline to the last visit (*P* = 0.344^c^). **E** The number of eyes occurred adverse events (*P* = 0.313^a^). ^a^ *P* value with Chi-square test; ^b^ *P* value with Fisher exact test; ^c^ *P* value with Mann–Whitney test; no significance (ns). DRSS, diabetic retinopathy severity scale; NV, neovascularization; BCVA, best-corrected visual acuity; CMT, central macular thickness; AEs, adverse events

Considering different intervals between IVC and PRP, the different strategies of combination therapy were analyzed (Fig. 2). 32 eyes and 29 eyes received IVC within (≤ 1 month group) and over a month after PRP (> 1 month group) and 17 eyes and 10 eyes were diagnosed as PDR in two group, respectively. There was no significant difference of age (*P* = 0.851), gender (*P* = 0.260), the severity of DR (*P* = 0.143) and occurrence of DME (*P* = 0.266) at baseline between two groups. After combination therapy, improvement in DRSS levels ≥ 2 steps was 14 eyes (44%) and 13 eyes (45%) in two groups (*P* = 0.933). The proportion of PDR patients with regression of NV after the combination treatment was not significantly different between two groups (*P* = 0.236), although the number was higher in ≤ 1 month group, 59% versus 30% in > 1 month group. There was also no significant difference between two groups in the change of BCVA (*P* = 0.078) and CMT (*P* = 0.334). The occurrence of adverse events was similar in both groups (*P* = 0.313).

### Risk factors for poor reactivity and adverse events

For patients with poor reactivity (improvement < 2 steps in the DRSS levels) and presence of vision-threatening adverse events (including vitreous or preretinal hemorrhage, vitrectomy and tractional retinal detachment) after combination treatment, risk factors were explored. Age, gender, BMI, history of diabetes, mean arterial pressure (MAP), biochemical indicators, DRSS levels and presence of DME at baseline, numbers of conbercept injections and the interval time between PRP and IVC were all included for analysis (Table 6, 7). Univariate analysis revealed that duration of diabetes, glomerular filtration rate (GFR), baseline DRSS levels, presence of DME and numbers of conbercept injections were significantly associated with poor reactivity to treatment. There was a close connection between DME and the number of conbercept injections based on clinical experience and correlation analysis (*P* < 0.001), so only the number of conbercept injections was included in multivariable analysis. For occurrence of adverse events, duration of diabetes and baseline DRSS levels were singled out. After incorporating these significant variables into multivariable logistic regression model, short diabetes duration (OR 0.849, 95%CI 0.734–0.982, *P* = 0.027), low GFR (OR 0.961, 95%CI 0.933–0.990, *P* = 0.010) and high baseline DRSS levels (OR 3.290, 95%CI 1.483–7.295, *P* = 0.003) were proved to be independent risk factors for poor reactivity to treatment. The presence of adverse events was mainly due to high DRSS levels at baseline (OR 3.668, 95%CI 1.710–7.868, *P* = 0.001).

**Table 6:** Univariate and multivariate logistic regression analysis of risk factors for poor reactivity to combination therapy

Characteristics	Univariate analysis			Multivariate analysis		
	OR	95%CI	*P* value	OR	95%CI	*P* value
Age	0.980	0.940–1.022	0.354	-	-	-
Gender	0.462	0.147–1.444	0.184	-	-	-
BMI	1.084	0.921–1.276	0.331	-	-	-
Diabetes type	1.28	0.168–9.732	0.811	-	-	-
Duration of diabetes	0.908	0.829–0.995	0.039*	0.849	0.734–0.982	0.027*
Family history of diabetes	0.616	0.191–1.988	0.417	-	-	-
MAP	0.996	0.963–1.030	0.821	-	-	-
HbA1c	0.908	0.655–1.259	0.564	-	-	-
TC	1.415	0.915–2.189	0.119	-	-	-
HDL	3.141	0.588–16.786	0.181	-	-	-
LDL	1.364	0.801–2.324	0.253	-	-	-
TG	1.143	0.673–1.943	0.621	-	-	-
ALT	0.99	0.947–1.035	0.663	-	-	-
AST	0.995	0.929–1.065	0.884	-	-	-
ALP	0.99	0.972–1.008	0.279	-	-	-
GFR	0.983	0.968–0.999	0.037*	0.961	0.933–0.990	0.010*
DRSS levels at baseline	2.881	1.508–5.506	0.001**	3.290	1.483–7.295	0.003**
Presence of DME at baseline	0.229	0.057–0.921	0.038*	-	-	-
Numbers of conbercept injections	0.675	0.492–0.926	0.015*	0.659	0.407–1.067	0.090
The interval between PRP and IVC	0.933	0.348–2.633	0.957	-	-	-

*OR* odds ratio, *CI* confidence interval, *BMI* body mass index, *MAP* mean arterial pressure, *HbA1c* glycated hemoglobin, *TC* total cholesterol, *HDL* high density lipoprotein, *LDL* low density lipoprotein, *TG* triglyceride, *ALT* glutamic pyruvic transaminase, *AST* glutamic oxaloacetic transaminase, *ALP* alkaline phosphatase, *GFR* glomerular filtration rate, *DRSS* diabetic retinopathy severity scale, *DME* diabetic macular edema, *PRP* panretinal photocoagulation, *IVC* intravitreal conbercept

^*^ *P* < 0.05, ** *P* < 0.01, *** *P* < 0.001

**Table 7:** Univariate and multivariate logistic regression analysis of risk factors for presence of vision-threatening adverse events

Characteristics	Univariate analysis			Multivariate analysis		
	OR	95%CI	*P* value	OR	95%CI	*P* value
Age	0.974	0.926–1.024	0.297	-	-	-
Gender	1.892	0.461–7.767	0.376	-	-	-
BMI	1.212	0.999–1.471	0.052	-	-	-
Diabetes type	-	-	0.999	-	-	-
Duration of diabetes	0.880	0.788–0.984	0.025*	0.905	0.794–1.032	0.137
Family history of diabetes	0.181	0.022–1.522	0.116	-	-	-
MAP	0.992	0.953–1.032	0.677	-	-	-
HbA1c	1.048	0.707–1.555	0.814	-	-	-
TC	1.381	0.871–2.192	0.17	-	-	-
HDL	0.458	0.064–3.272	0.437	-	-	-
LDL	1.62	0.883–2.971	0.119	-	-	-
TG	1.113	0.618–2.003	0.721	-	-	-
ALT	1.011	0.963–1.062	0.657	-	-	-
AST	1.008	0.931–1.092	0.836	-	-	-
ALP	1.004	0.984–1.023	0.724	-	-	-
GFR	0.996	0.979–1.013	0.633	-	-	-
DRSS levels at baseline	3.868	1.847–8.102	< 0.001***	3.668	1.710–7.868	0.001**
Presence of DME at baseline	0.316	0.087–1.140	0.078	-	-	-
Numbers of conbercept injections	0.837	0.609–1.151	0.274	-	-	-
The interval between PRP and IVC	0.532	0.155–1.828	0.317	-	-	-

*OR* odds ratio, *CI* confidence interval, *BMI* body mass index, *MAP* mean arterial pressure, *HbA1c* glycated hemoglobin, *TC* total cholesterol, *HDL* high density lipoprotein, *LDL* low density lipoprotein, *TG* triglyceride, *ALT* glutamic pyruvic transaminase, *AST* glutamic oxaloacetic transaminase, *ALP* alkaline phosphatase, *GFR* glomerular filtration rate, *DRSS* diabetic retinopathy severity scale, *DME* diabetic macular edema, *PRP* panretinal photocoagulation, *IVC* intravitreal conbercept

^*^ *P* < 0.05, ** *P* < 0.01, *** *P* < 0.001

### Representative cases

**Fig. 3:**
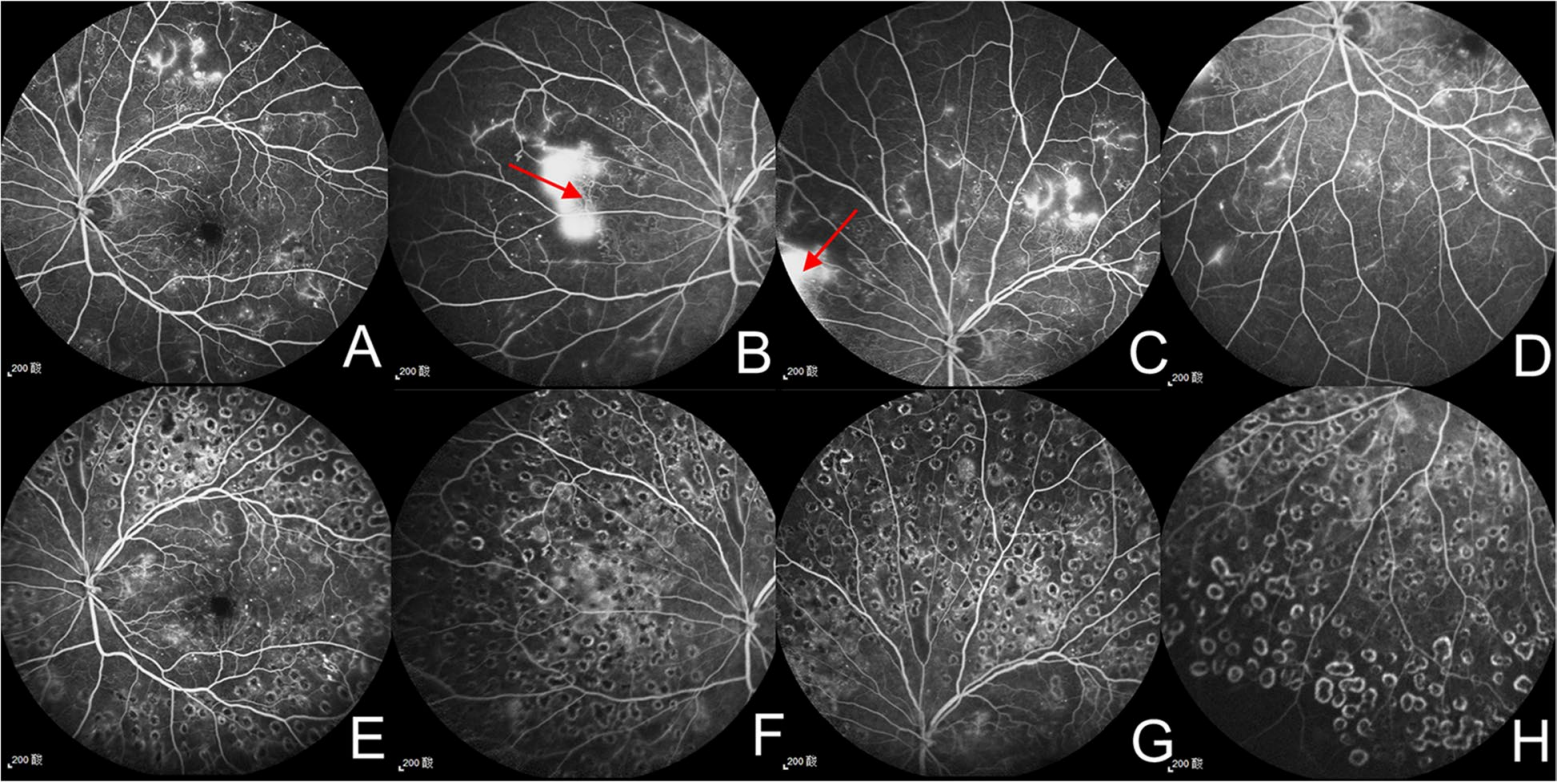
Patients with better reactivity after combination treatment. **A**-**D** The patients (left eye) with DRSS level 61 (mild PDR) at baseline. FFA shows NVE with hyperfluorescence (red arrows). **E**–**H** Over 6 months after PRP and one IVC injection. FFA shows improvement in DRSS with totally regression of NVE. DRSS, diabetic retinopathy severity scale; FFA, fundus fluorescein angiography; NVE, neovascularization elsewhere; PRP, panretinal photocoagulation; IVC, intravitreal conbercept

Case 1 A 56-year-old man had 20-year history of diabetes with GFR of 105.82 ml/min/1.73m^2^. FFA showed NVE in nasal quadrant and DRSS level was 61 (mild PDR) before treatment (Fig. 3A-D). After given PRP and one IVC injection, NVE regressed totally as shown in FFA over 6 months after treatment (Fig. 3E–H).

**Fig. 4:**
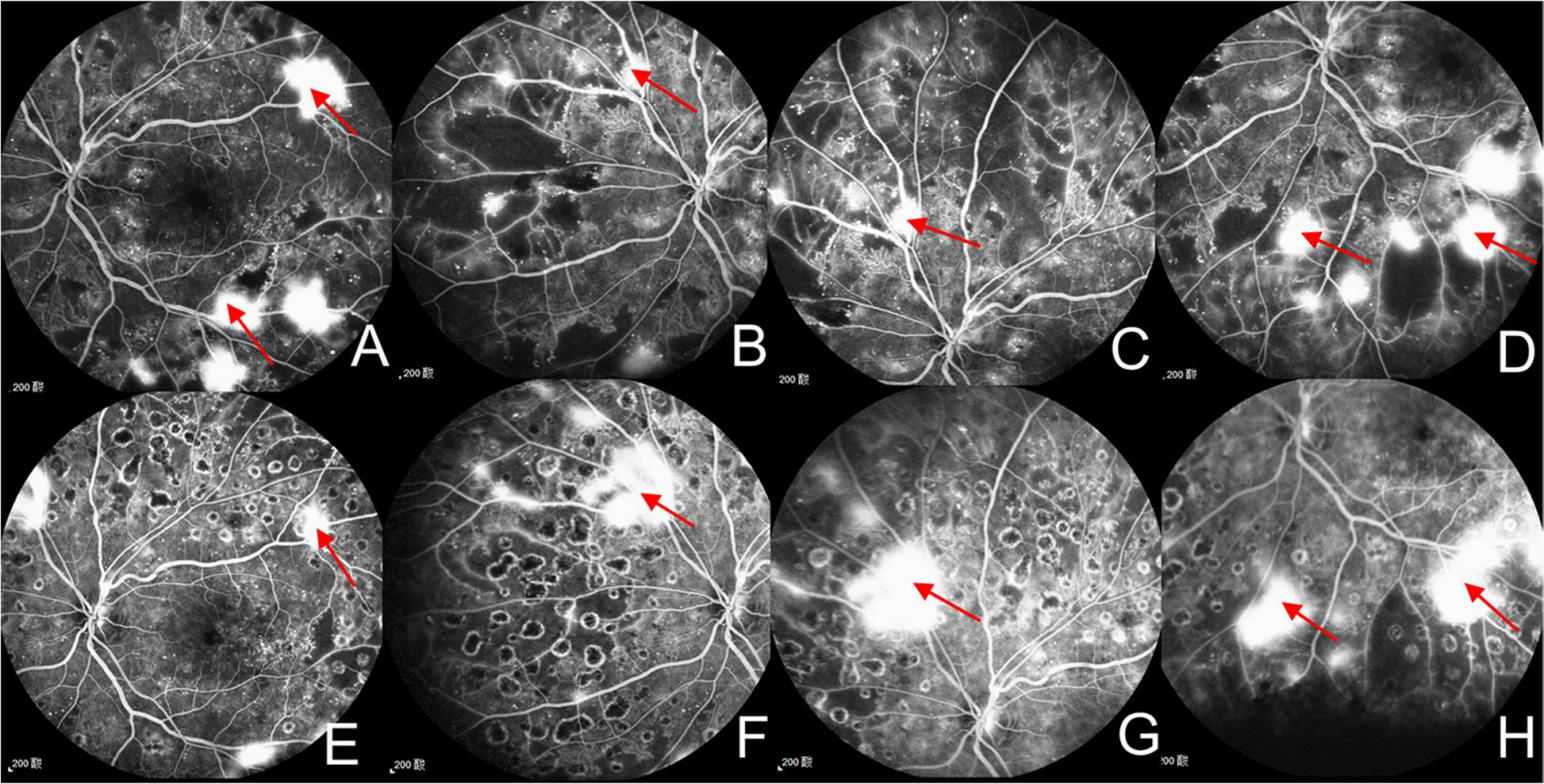
Patients with poor reactivity after combination treatment. **A**-**D** The patients (left eye) with DRSS level 65 (moderate PDR) at baseline. FFA shows NVE > 0.5 DA in 1 + quadrants (red arrows). **E**–**H** Over 6 months after PRP and five IVC injections. NVE still had no regression totally and DRSS level was still 65. DRSS, diabetic retinopathy severity scale; FFA, fundus fluorescein angiography; NVE, neovascularization elsewhere; DA, disc area; PRP, panretinal photocoagulation; IVC, intravitreal conbercept

Case 2 A 54-year-old man had only 5-year history of diabetes with GFR of 95.75 ml/min/1.73m^2^. FFA showed lager NVE and nonperfusion areas in all 4 quadrants with DRSS level 65 (moderate PDR) before treatment (Fig. 4A-D). After given PRP and five IVC injections, nonperfusion areas decreased but NVE still existed over 6 months after treatment as shown in FFA (Fig. 4E–H).

**Fig. 5:**
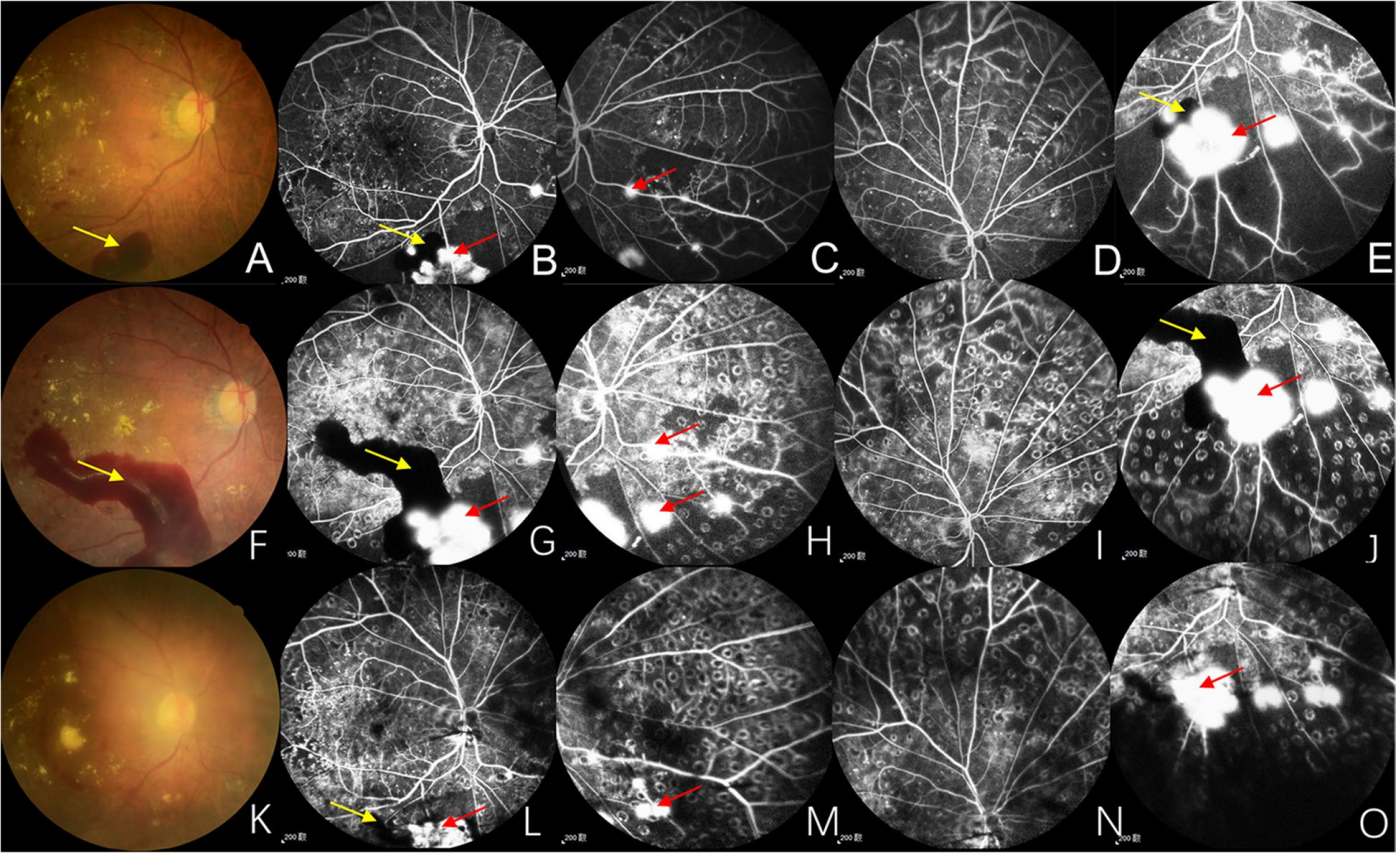
Patients with adverse events after combination therapy. **A**-**E** The patients (right eye) with DRSS level 71,75 (high-risk PDR) at baseline. FP and FFA shows NVE > 0.5 DA in 1 + quadrants (red arrows) with PRH (yellow arrows). **F**-**J** Over 6 months after PRP and one IVC injection. PRH increased and NVE still had no regression. **K**–**O** After given five additional IVC injections, PRH absorbed gradually but NVE still had no regression totally over one and a half years. DRSS, diabetic retinopathy severity scale; FP, fundus photography; FFA, fundus fluorescein angiography; NVE, neovascularization elsewhere; DA, disc area; PRP, panretinal photocoagulation; IVC, intravitreal conbercept; PRH, preretinal hemorrhage

Case 3 A 63-year-old man had a history of diabetes for ten years. The DRSS level at baseline was 71,75 (high-risk PDR) with large NVE and preretinal hemorrhage (PRH) (Fig. 5A-E). After treated with PRP and one IVC injection, NVE had no obvious change and PRH increased after 6 months (Fig. 5F-J). Then five IVC injections were given additionally, PRH absorbed gradually but NVE still had no regression totally over one and a half years (Fig. 5K–O).

## Discussion

After becoming the first-line treatment for DME, the validation of anti-VEGF therapy in DR was also confirmed [16–18]. It was reported that intravitreal anti-VEGF could reduce the risk of developing to vision-impairing complications [19]. Subsequently, some studies showed the effectiveness of VEGF as an adjunct therapy to PRP for the treatment of PDR [12, 20, 21]. The PROTEUS study enhanced the result and demonstrated the efficacy of the combined treatment for regression of NV compared to PRP individually. Thus, we retrospectively summarized the results of combination therapy for patients with DR in real world.

Compared to three injections at loading phase in other studies, patients only received intravitreal anti-VEGF therapy as need based on presence of NV or investigator evaluation in our study which was close to a real-world setting. Therefore, 48% of patients with PDR at baseline presented NV regression, which was lower than above 90% of NV regression reported in other studies [11, 21, 22]. However, patients in these studies were given a median of 6 anti-VEGF injections and followed up strictly which was difficult to achieve in real-world setting. Instead of ranibizumab widely used in other researches, patients in our study treated with conbercept which had a lower VEGF dissociation rate, higher binding affinity and longer clearance time [23]. We also included some NPDR patients with DRSS levels of 47–53 and put the improvement of DRSS levels as the main outcome rather than NV reduction. Compared to PDR group, the patients with NPDR had a higher improvement in DRSS levels after combination therapy, as well as lower occurrence of adverse events. A binary logistic regression analysis supported the result and showed that high levels of DRSS at baseline was an independent risk factor of DRSS improvement less than 2 levels and occurrence of adverse events. Thus, combination therapy should be given to the patients at earlier stage of DR.

For different strategies of combination therapy, there is no consensus currently and few studies mentioned it. Zhang et al. reported that there was no significant difference in change of BCVA and CMT between IVC before and after PRP group. For IVC after PRP therapy, the result of subgroup analysis in our study showed that eyes received PRP combined with earlier (≤ 1 month) or later (> 1 month) anti-VEGF therapy were also no significant difference for outcomes [24].

A longer duration of diabetes, higher HbA1c level, hypertension, and hyperlipidemia were risk factors for the presence of DR as the most commonly reported [25, 26]. But very few studies noticed factors related to prognosis of DR after treatment. We evaluated the potential indictors which might lead to different outcomes after combination therapy. A binary logistic regression analysis revealed high baseline DRSS levels, short duration of diabetes and low GFR as independent risk factors for poor response to treatment. DRSS level represented the severity of DR which had a high proportion for different respond of combination therapy among these three factors. Patients with more severe DR are more likely to develop complications in a short time. Besides, the result of our study showed that the occurrence of adverse events was only related to DRSS levels at baseline. It suggested that combination therapy does not completely prevent the occurrence of VTDR, especially for patients with high baseline DRSS levels. Thus, earlier intervention to patients with a lower DRSS levels may obtain a better prognosis. Duration of diabetes symbolized the rate of progression of DR. It had a higher speed to develop the same DRSS level for the patients with a shorter diabetes duration compared to the longer one, which may indicate that they had a poor respond to the combination therapy, so patients who appeared DR with short duration of diabetes were tend to have a rapid progression of DR. It reminded us that we should focus on that developed DR at the early stage of diabetes who were more likely to have a poor prognosis. GFR was a factor related to renal function which indicated nephropathy caused by diabetes. Recently, Zhao et al. mentioned the significant correlation between renal function and the development of DR [27]. They found that GFR decreased with the progression of moderate to proliferative DR, which may due to the similar microvascular pathophysiologic mechanisms between DR and diabetic nephropathy [28]. The level of GFR may indirectly represent the development of DR and the patients with impaired GFR were tended to have poor response to treatment. Therefore, more attentions should be paid to patients with high DRSS levels, poor renal function and occurred DR at the early stage of diabetes.

The main limitation of this study was the retrospective nature and patients with incomplete information and follow-up time less than 6 months were excluded although they received combination therapy. Thus, a selection bias existed in our study and the result in our study cannot fully represent the outcomes of combination therapy for DR. Further studies with prospective property are needed to confirm the outcomes. Another limitation was the number of patients. A larger size sample would be more ideal to verify our results as well as to select the risk factor for patients with adverse events.

In conclusion, our study showed that PRP plus IVC was effective for most patients with DR and there was no significant difference for eyes with earlier or later IVC in a real-world setting. Poor renal function, high DRSS levels or occurred DR at the early stage of diabetes were demonstrated to be the independent risk factors for patients with poor response to treatment and the occurrence of adverse events was only related to DRSS levels at baseline.

## Data Availability

The datasets used and analyzed during the current study are available from the corresponding author on reasonable request.

## Abbreviations

AEs: Adverse events
ALT: Glutamic pyruvic transaminase
AST: Glutamic oxaloacetic transaminase
ALP: Alkaline phosphatase
BCVA: Best-corrected visual acuity
BMI: Body mass index
CMT: Central macular thickness
CI: Confidence interval
DA: Disc area
DME: Diabetic macular edema
DRSS: Diabetic retinopathy severity scale
FFA: Fundus fluorescein angiography
FP: Fundus photography
GFR: Glomerular filtration rate
HbA1c: Glycated hemoglobin
HDL: High density lipoprotein
IOP: Intraocular pressure
IRMA: Intraretinal microvascular abnormality
IVC: Intravitreal conbercept
LDL: Low density lipoprotein
MAP: Mean arterial pressure
NPDR: Nonproliferative diabetic retinopathy
NV: Neovascularization
NVD: Neovascularization of the disc
NVE: Neovascularization elsewhere
OR: Odds ratio
PDR: Proliferative diabetic retinopathy
RH: Retinal hemorrhage
PRH: Preretinal hemorrhage
PRP: Photocoagulation
TC: Total cholesterol
TG: Triglyceride
TRD: Tractional retinal detachment
VEGF: Vascular endothelial growth factor
VH: Vitreous hemorrhage
